# HIV and Syphilis Testing Among Patients Tested for Gonorrhea and Chlamydia in Emergency Departments

**DOI:** 10.5811/westjem.48813

**Published:** 2026-04-21

**Authors:** Kyla Sherwood, Neil Zhang, Hollie David, Annette Dekker, Omai Garner, Elizabeth Samuels, Paul Adamson

**Affiliations:** * University of California, Los Angeles, Division of Preventive Medicine, Department of Medicine, Los Angeles, California; † Cedars Sinai Medical Center, Division of Cardiology, Department of Medicine,Los Angeles, California; ‡ University of California, Los Angeles, Division of Infectious Diseases, Department of Medicine, Los Angeles, California; § University of California, Los Angeles, Department of Emergency Medicine, Los Angeles, California; || UCLA Medical Center, Department of Pathology and Laboratory Medicine, UCLA Medical Center, Los Angeles, California

## Abstract

**Introduction:**

Sexually transmitted infections (STIs), including HIV and syphilis, are increasing. In 2023, there were over 2.4 million reported cases of chlamydia, gonorrhea, and syphilis in the United States, a 32.5% increase from 2014. Emergency departments (EDs) are vital touchpoints for STI testing, yet HIV and syphilis testing among patients undergoing Neisseria gonorrhoeae (NG) and Chlamydia trachomatis (CT) testing is suboptimal. We aimed to determine testing frequency and to identify factors associated with HIV and syphilis co-testing among ED patients undergoing NG/CT testing.

**Methods:**

We conducted a retrospective observational study of all patients tested for NG/CT from 2021–2024 at two Los Angeles EDs. Covariates including sociodemographic and behavioral data were extracted from the medical record. The primary outcome was complete STI testing, defined as both HIV and syphilis testing during or up to six months prior to an ED encounter with NG/CT testing. Multivariable logistic regression was used to evaluate factors associated with complete STI testing.

**Results:**

Of 3,940 patients, 459 (11.7%) received complete STI testing. Among patients receiving complete STI testing, 176 (38.3%) were female, 282 (61.4%) were male, 96 (20.9%) were Hispanic, 98 (21.4%) were non-Hispanic Black, 195 (42.5%) were non-Hispanic White, 220 (47.9%) had Medicare insurance, 132 (28.8%) had private insurance, 225 (49.0%) were experiencing homelessness, 14 (3.1%) identified as bisexual, and 90 (19.6%) identified as heterosexual. In multivariable analysis, patients who were bisexual (adjusted odds ratio [aOR] 2.51; 95% CI, 1.32–4.80; P = .005); had Medicare insurance (aOR 1.89; 1.20–2.98; P = .006); or were experiencing homelessness (aOR 5.21; 4.00–6.78; P < .001) had higher odds of complete STI testing. Patients who were Hispanic (aOR 0.69; 0.52–0.92; P = .01); non-Hispanic Black (aOR 0.75; 0.56–1.00, P = .05); or female (aOR 0.68; 0.54–0.85; P = .001) had lower odds. Of 261 patients with multiple ED encounters, 217 (83.1%) never received complete testing.

**Conclusion:**

Complete HIV and syphilis testing among ED patients tested for N. gonorrhoeae and C. trachomatis was low, even among patients with multiple ED encounters. Lower testing among Hispanic and non-Hispanic Black patients may exacerbate existing disparities in STIs. Implementation research is needed to improve the integration of STI testing in EDs.

## INTRODUCTION

National rates of sexually transmitted infections (STIs) have increased significantly over the past decade with persistent racial disparities.^1^ In 2023, there were over 2.4 million reported cases of chlamydia, gonorrhea, and syphilis in the United States, representing a 32.5% increase from 2014.^1^ Nationwide efforts, including the expansion of the U.S. Preventive Services Task Force HIV screening guidelines and the creation of national programs such as the Ending the HIV Epidemic (EHE) program and the National Syphilis and Congenital Syphilis Syndemic Federal Task Force, were created to address these growing epidemics.^2^,^3^ Yet priority jurisdictions targeted by these initiatives, such as Los Angeles County in Southern California, continue to experience dramatic increases in STI rates.^2^,^3^ Approximately 59,400 people live with HIV in Los Angeles County, and there are 1,518 new diagnoses annually. In 2023, there were 4,897 newly reported cases of early syphilis (53 per 100,000 people).^4^,^5^ The majority of new HIV diagnoses occurred in men, particularly men who have sex with men, and higher HIV diagnosis rates were seen in non-Hispanic Black and Hispanic populations.^6^

Emergency departments (EDs) represent critical sites for screening and care delivery for patients with syphilis and HIV, particularly among underserved populations who may otherwise lack access to care.^7^,^8^ EDs in EHE-priority jurisdictions disproportionately serve patients with higher rates of bacterial STIs and limited insurance coverage.^7^–^9^ These EDs also more frequently care for populations prioritized by the EHE initiative for outreach efforts (eg, non-Hispanic Black and Hispanic patient populations) and have been identified as key settings for expanded HIV testing efforts.^7^,^9^

Gonorrhea and chlamydia are important risk factors for HIV and syphilis acquisition, with high rates of coinfection.^10^, ^23^,^24^ Accordingly, current STI guidelines recommend HIV and syphilis screening for all patients tested for *Neisseria gonorrhoeae* (NG) and *Chlamydia trachomatis* (CT). Yet, prior ED-based studies have demonstrated consistently low rates of concurrent HIV and syphilis testing among patients tested for NG/CT, ranging from < 1 to 30%.^11^–^14^,^23^–^25^ Despite this, factors associated with incomplete STI testing in the ED remain poorly understood. Low rates of complete STI testing may result in missed HIV and syphilis diagnoses, increasing patients’ risk of untreated infection-related complications and contributing to ongoing transmission in communities.^15^

In this retrospective observational study, we aimed to characterize HIV and syphilis testing among ED patients tested for NG/CT in an EHE-priority jurisdiction and to identify factors associated with incomplete testing. By characterizing patterns of testing and identifying missed screening opportunities, we sought to inform future implementation strategies to improve complete STI screening in the ED setting.

**Population Health Research Capsule**

What do we already know about this issue?

*The incidence of sexually transmitted infections (STI) is rising nationwide with persistent racial disparities. Concurrent HIV and syphilis testing remains low*.

What was the research question?

*We aimed to identify factors associated with missed HIV and syphilis testing to improve ED-based STI screening*.

What was the major finding of the study?

*Hispanic (aOR 0.69, CI 0.52–0.92), Black (0.75, 0.56–1.00), and female (0.68, 0.54–0.85) patients had lower odds of complete STI testing*.

How does this improve population health?

*Our study underscores the need to expand ED-based STI testing, prioritize vulnerable populations, and implement targeted ED interventions and clinician education*.

## METHODS

### Study Design

This was a retrospective, observational study of all patients ≥ 13 years of age who received NG/CT testing from January 1, 2021–June 30, 2024 at two large, urban EDs in Los Angeles. One ED represents a community hospital staffed by attending physicians, and the second a quaternary, academic referral center staffed by attending physicians and medical trainees. During the study period, no standardized protocol existed for syphilis and HIV testing beyond routine clinical care, and STI testing was conducted based on clinicians’ clinical judgment.

### Data Collection

Data were extracted through an automated query of the electronic health record (EHR) for all patients who received NG/CT testing (including urine, pharyngeal, vaginal, urethral, or rectal specimens) during an ED encounter (prior to ED discharge or hospital admission) within the study period. The STI testing was available to be performed at both EDs at any time of day.

We extracted HIV and syphilis testing data during or up to six months prior to the ED encounter. Prior testing was included because clinicians may not repeat testing if it had recently occurred. Both EDs used HIV testing via fourth-generation HIV antigen/antibody tests and a traditional syphilis testing algorithm (rapid plasma reagin (RPR) test with confirmatory *Treponema pallidum* particle agglutination when requested). Syphilis positivity was defined as an RPR titer ≥ 1:8.^16^ HIV viral load testing during or up to six months prior to the ED encounter was performed using HIV RNA polymerase chain reaction testing. In both EDs, all patients with positive test results that returned after an ED encounter were called by an attending physician to notify them of the test result; positive STI tests followed this same procedure. Treatment was offered with antibiotics for positive syphilis, chlamydia, or gonorrhea testing, and patients were offered referrals and/or additional resources to establish HIV care.

Patient demographics included age, sex, race and ethnicity, sexual orientation, health insurance, and history of homelessness. Sex was categorized as male, female, and unknown. Race and ethnicity were categorized as Asian, Hispanic, non-Hispanic Black (hereafter, Black), non-Hispanic White (hereafter, White), other, and unknown. “Other race” included American Indian or Alaska Native, Native Hawaiian or other Pacific Islander, or responses of “other race,” and were combined due to overall small patient numbers. Patients were categorized as unknown race if their EHR intake response was “decline to answer,” “does not identify with race,” or there were missing responses. Sexual orientation was categorized as heterosexual, bisexual, lesbian or gay, other sexual orientation, or unknown.

All patient demographic data were self-reported and collected from the EHR. Health insurance was categorized as private insurance, Medicare, Medicaid, and other. History of homelessness was defined if it was present in the problem list or if a residential address was missing. We grouped data into half-year periods from January 1–June 30 and July 1–December 31 to provide sufficient patient numbers to evaluate temporal trends. These variables were included in our multivariable regression based on our conceptual model for complete STI screening in the ED setting.

### Outcomes

The primary outcome was complete STI testing, which was defined as obtaining both HIV and syphilis testing during or up to six months prior to the ED encounter in which a patient received NG/CT testing. Incomplete STI testing was defined as missing HIV and/or syphilis testing during and up to six months prior to the ED encounter.

### Data Analyses

We performed descriptive statistics to evaluate patient characteristics with complete as compared to incomplete STI testing. Multivariable logistic regression was performed to evaluate factors associated with complete as compared to incomplete STI testing, specifically evaluating the variables of age, sex, race and ethnicity, sexual orientation, health insurance, history of homelessness, and half-year time period. Interactions were assessed between sex, race and ethnicity, and experiencing homelessness. For the primary analysis, we excluded repeat ED encounters by the same patient. A separate analysis was performed for patients with multiple ED encounters. We used Pearson chi-squared testing to evaluate trends in STI testing and positivity over the study period. Data analysis was conducted in STATA v18.0 (StataCorp, LLC, College Station, TX). All statistical tests were two-sided. For all statistical testing comparing complete vs incomplete STI testing, significance was set at P < 0.05.

This study was reviewed by the UCLA Institutional Review Board (IRB) with a waiver for informed consent. Analysis and manuscript preparation followed STROBE guidelines (Appendix). This study followed 9 of 12 method criteria outlined by Worster et al: case selection criteria; variable definition; use of abstraction forms; performance monitored; blind to hypothesis; medical records identified; sampling method; missing-data management plan; and IRB approval.^17^

## RESULTS

A total of 3,940 patients received NG/CT testing during the study period, of whom 2,101 (53.3%) were female, 1,437 (36.5%) were White, 1,196 (30.4%) identified as heterosexual, and 1,746 (44.3%) had private health insurance (Table 1).

Complete STI testing was performed in 459 (11.7%) patients. Patients with complete STI testing were more likely to be older, male, and experiencing homelessness (all *P* < .001). Of the 3,481 patients with incomplete STI testing, 2,980 (85.6%) patients received neither HIV nor syphilis testing, 227 (6.5%) received HIV testing, and 274 (7.8%) received syphilis testing.

### Factors Associated with Complete Testing

**Figure 1:**
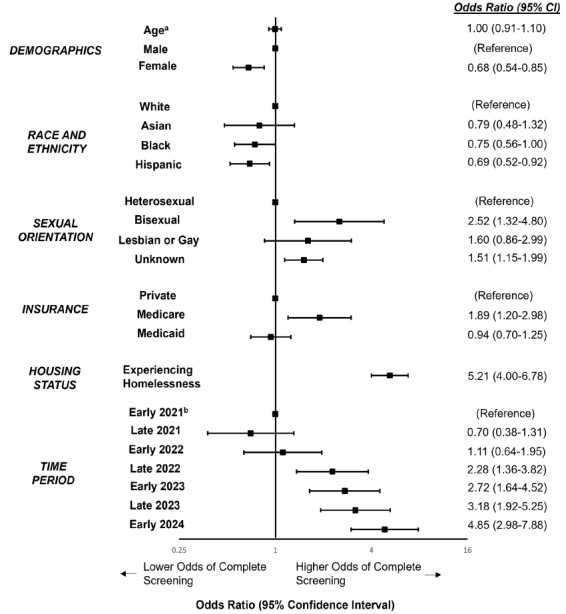
Forest plot depicts the adjusted odds ratio estimates of patient and encounter factors associated with receiving complete HIV and syphilis testing in the multivariable logistic regression model. ^a^Age per 10 years. ^b^Early refers to January–June. Late refers to July–December.

In the multivariable analysis, patients who were Hispanic (adjusted odds ratio [aOR] 0.69; 95% CI, 0.52–0.92), Black (aOR 0.75; 0.56–1.00, *P* = .05), and female (aOR 0.68; 0.54–0.85) had lower odds of complete STI testing. Patients who were bisexual (aOR 2.51; 1.32–4.80), had Medicare insurance (aOR 1.89; 1.20–2.98) and were experiencing homelessness (aOR 5.21; 4.00–6.78) had higher odds of complete STI testing (Figure 1). There were no significant interactions between sex and race. As compared to those who were non-Black and housed, Black patients experiencing homelessness had 48% lower odds of receiving complete testing (aOR 0.52; 0.31–0.88).

### Multiple Emergency Department Encounters

There were 261 (6.6%) patients with ≥ 2 ED encounters. Patients with multiple ED encounters were significantly more likely and be Black (*P* < .001). Of these 261 patients, 25 (9.6%) patients received complete STI testing during their first encounter, an additional 19 (8.1%) patients received complete testing during their second encounter, and no additional patients received testing during their third encounter.

### Temporal Trends

**Figure 2:**
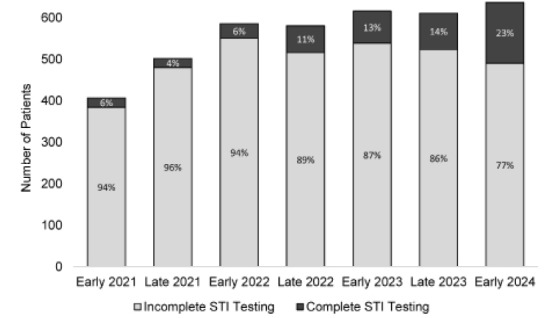
Number and proportion of patients who received complete and incomplete HIV and syphilis testing by half-year period (2021–2024) in a study identifying factors related to incomplete vs complete HIV and syphilis testing in the emergency department. *Early refers to January–June. Late refers to July–December. *STI*, sexually transmitted infection.

**Figure 3:**
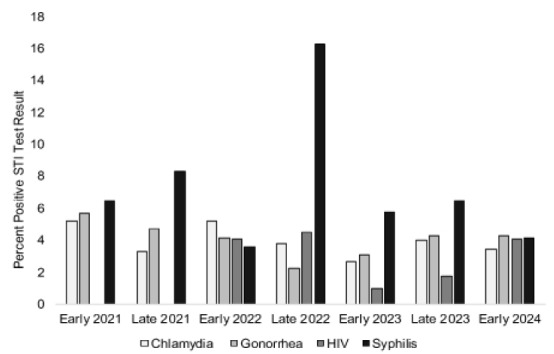
Proportion with positive test results for gonorrhea, chlamydia, HIV, and syphilis by half-year period (2021–2024) in a study identifying factors related to incomplete vs complete HIV and syphilis testing in the emergency department. *Early refers to January–June. Late refers to July–December. *STI*, sexually transmitted infection.

Analysis of temporal trends in STI testing revealed that the total number of patients with NG/CT testing increased over the study period, as did the proportion of patients receiving complete STI testing starting in 2022 (*P* < .001) (Figure 2). The STI positivity rates were not found to be significantly different over the study period except for syphilis positivity (Figure 3).

## DISCUSSION

We found that fewer than 12% of patients undergoing NG/CT testing received HIV and syphilis testing. Black, Hispanic, and female patients were less likely to receive complete HIV and syphilis testing in the ED. Those experiencing homelessness and who identified as bisexual were more likely to receive complete testing. Over 83% of patients with repeated ED encounters with NG/CT testing never received syphilis and HIV testing. These findings highlight critical gaps in ED-based STI testing and underscore the need for strategies to improve comprehensive ED-based STI testing.

Many factors may contribute to low complete STI testing, including the fast-paced ED environment, patient factors, clinician knowledge and/or clinical priorities, and the logistics of obtaining STI test results.^14^ Both syphilis and HIV testing require blood draws, which may limit ease of testing compared to NG/CT swabs. Another consideration is that while syphilis, gonorrhea, and chlamydia can be treated with either intramuscular or oral antibiotics, new HIV diagnoses require establishing longitudinal HIV care. We recognize that ED clinicians may be hesitant to offer HIV testing due to concerns about optimally addressing new HIV diagnoses from the ED setting. However, most patients with incomplete testing did not receive either HIV or syphilis testing, and more patients missed syphilis as compared to HIV testing (274 vs 227 patients, respectively). Thus, concerns about longitudinal follow-up of positive HIV test results did not clearly appear to drive incomplete STI testing. Notably, NG/CT testing volumes were also low in this ED setting. Across the study period, an average of three patients per day received NG/CT testing at both ED sites, further underscoring the low utilization of ED-based STI screening.

The racial disparities in HIV and syphilis testing observed in our study are concerning as they may compound existing STI disparities. Our finding that Black and Hispanic patients were less likely to receive complete syphilis and HIV testing is consistent with prior literature.^14^,^18^ In those reports, lower complete STI testing may have been influenced by bias, stigma, or patient distrust of the medical system.^14^,^18^ In this study, we were unable to assess the reasons for incomplete testing in these populations. Nationwide, higher rates of syphilis and HIV occur among Black and Hispanic populations.^1^ In Los Angeles County, Black patients experienced disproportionately higher rates of HIV diagnoses, representing 22% of HIV diagnoses, despite accounting for only 8% of the population, and Hispanic mothers represented 64% of all congenital syphilis cases.^6^,^19^ We propose that focused efforts to prioritize STI testing are critical in addressing these persistent racial STI disparities in these populations.

Our findings underscore how social determinants of health may influence the likelihood of complete HIV and syphilis testing. Experiencing homelessness was associated with a 5-fold increase in the odds of complete testing. To our knowledge, housing status has not been evaluated in prior ED STI literature. We hypothesize that clinicians recognize that people experiencing homelessness represent a vulnerable population that has unique barriers to care and markedly benefit from ED-based comprehensive STI screening.^20^ However, racial disparities persisted even among those experiencing homelessness, as Black patients experiencing homelessness were less likely to receive complete testing.

Our finding that most patients with multiple ED presentations did not receive complete STI testing was particularly concerning. Patients with repeated ED encounters involving NG/CT testing represent a population at elevated risk for HIV and syphilis, given the high prevalence of STI coinfections and the well-established role of gonorrhea and chlamydia as risk factors for HIV acquisition.^10^, ^23^, ^24^ Recurrent ED encounters with NG/CT testing may also reflect limited access to longitudinal primary care, representing a patient population that disproportionately relies on ED-based care for STI testing. Together, these findings suggest that patients with multiple ED encounters constitute a population at increased risk for HIV and syphilis for whom testing was repeatedly missed despite multiple opportunities.

Notably, we observed an increase in the proportion of patients with complete STI testing throughout the study period, which did not appear to be related to changes in patient demographics. Several possible explanations may account for this observation. First, clinical disruptions during the COVID-19 pandemic may have limited or de-emphasized ED-based STI testing in the early study period, which improved over time. Second, growing awareness of the HIV and syphilis epidemics—possibly driven by public health campaigns—may have increased clinicians’ recognition of the importance of STI testing. Although further research is needed to clarify the specific factors underlying this trend, EHE-priority jurisdictions have documented increases in HIV testing, indicating that outreach efforts may be improving HIV testing practices.^21^ However, more than 75% of patients still did not receive complete testing at the end of the study period, underscoring the ongoing need for innovation to improve ED-based STI testing.

## LIMITATIONS

There were several limitations to this study. Incomplete testing may relate to patient preference, which was not collected for this study. However, previous studies have demonstrated that patients undergoing screening prefer testing for all STIs rather than a subset.^22^ Patients may have also favored empiric treatment rather than testing for syphilis, which we were unable to determine. However, most patients did not receive both HIV and syphilis testing, suggesting empiric treatment was not the sole reason for incomplete STI testing. Given the retrospective nature of the study, we could not determine causality or directionality in the relationship between NG/CT and syphilis/HIV testing (ie, our study may have captured patients who were initially targeted for HIV/syphilis testing). Our findings are significant despite the inclusion of these patients, which would likely have biased the results toward the null. As a retrospective review, some demographic variables, particularly sexual orientation, were limited to what was reported in the EHR.

During the study period, there were changes within the EHR on how these data were collected. Nevertheless, sexual orientation was included in the model, given the well-established increased burden of STIs and HIV among the population of men who have sex with men. Due to limitations of our data extraction process and the sensitive nature of HIV diagnoses, it was not feasible to determine patients’ HIV statuses prior to their ED visit. However, it would be appropriate when performing NG/CT testing to send an isolated HIV viral load testing as part of an evaluation for patients known to be living with HIV; the HIV viral load test was only performed for 37 patients at the time of ED encounter or six months prior, with 24 patients having detectable viral loads. This finding suggests that patients known to be living with HIV likely did not represent a significant portion of the study population.

Lastly, this study occurred at two EDs within a single academic health system. While this may limit the generalizability, the study setting is significant given its location in an Ending the HIV Epidemic-priority county serving a diverse patient population.

## CONCLUSION

Despite increased awareness and national organizations targeting rising rates of HIV and syphilis through expanded screening guidelines for STIs and public health initiatives, comprehensive STI screening in ED settings remains an ongoing challenge.^2^,^3^ Our study highlights missed opportunities for HIV and syphilis testing among Black and Hispanic populations, who already suffer from disproportionately higher rates of HIV and syphilis. HIV and syphilis testing was also repeatedly missed among patients with multiple ED encounters for NG/CT testing.

Emergency departments are likely to play an increasingly vital role as the safety net for STI care in the United States. To effectively address gaps in ED-based STI testing, implementation research is needed to evaluate targeted interventions, including clinician education on STI prevalence, the frequency of coinfections, and optimal STI screening test ordering practices. Enhancements to the electronic health record, such as clinical decision support tools, may prompt clinicians to order complete STI testing through standardized order panels or best practice alerts.

Additionally, streamlining protocols to deliver rapid, actionable STI testing results prior to ED discharge, along with establishing durable referral pathways for the management of positive results, may further facilitate increased testing. These interventions will be evaluated in follow-up implementation work at our institution to generate additional insight and best practices for improving complete STI testing in the ED setting.

## Supplementary Information



## Figures and Tables

**Table 1:** Cohort characteristics in a study evaluating HIV and syphilis testing among patients tested for *Neisseria gonorrhoeae* and *Chlamydia trachomatis* in the emergency department.

	Total cohort (N = 3,940)	Incomplete testing (n = 3,481)	Complete testing (n = 459)	P value^a^
Mean Age (SD), year	36 (13)	35 (13)	39 (15)	< .001
Sex at Birth—no. (%)^b^				< .001
Female	2,101 (53.3%)	1,925 (55.3%)	176 (38.3%)	
Male	1,828 (46.4%)	1,546 (44.4%)	282 (61.4%)	
Unknown	11 (0.3%)	10 (0.3%)	1 (0.2%)	
Race and Ethnicity—no. (%)^c^				< .001
Asian	267 (6.8%)	247 (7.1%)	20 (4.4%)	
Black	705 (17.9%)	607 (17.4%)	98 (21.4%)	
Hispanic	1,154 (29.3%)	1,058 (30.4%)	96 (20.9%)	
Other	224 (5.7%)	192 (5.5%)	32 (7.0%)	
Unknown	153 (3.9%)	135 (3.9%)	18 (3.9%)	
White	1,437 (36.5%)	1,242 (35.7%)	195 (42.5%)	
Sexual Orientation—no. (%)^d^				<.001
Heterosexual	1,,196 (30.4%)	1,106 (31.8%)	90 (19.6%)	
Lesbian or gay	111 (2.8%)	96 (2.8%)	15 (3.3%)	
Bisexual	99 (2.5%)	85 (2.4%)	14 (3.1%)	
Other	51 (1.3%)	44 (1.3%)	7 (1.5%)	
Unknown	2,483 (63.0%)	2,150 (61.8%)	333 (72.5%)	
Experiencing Homelessness—no. (%)				< .001
No	3,252 (82.5%)	3,018 (86.7%)	234 (51.0%)	
Yes	688 (17.4%)	463 (13.3%)	225 (49.0%)	
Health Insurance—no. (%)^e^				< .001
Private	1,746 (44.3%)	1,614 (46.4%)	132 (28.8%)	
Medicaid	1,644 (41.7%)	1,424 (40.9%)	220 (47.9%)	
Medicare	224 (5.7%)	160 (4.5%)	64 (13.9%)	
Other	325 (8.3%)	282 (8.1%)	43 (9.4%)	

a Statistics were performed using t-tests and Pearson chi-squared tests for continuous and categorical data, respectively. P values are for statistical tests comparing complete testing to incomplete testing.

b Percentages represent column percent.

c Other under race and ethnicity includes American Indian or Alaska Native, Native Hawaiian or other Pacific Islander, or other race. Unknown under race and ethnicity includes responses of declined to answer or does not identify with race and missing responses.

d Other under sexual orientation includes responses of other or something else. Unknown under sexual orientation includes missing responses and responses of don’t know or choose not to disclose.

e Other under health insurance includes all health insurances besides private, Medicaid, and Medicare as well as any missing responses.
